# The amino acid metabolomics signature of differentiating myocardial infarction from strangulation death in mice models

**DOI:** 10.1038/s41598-023-41819-6

**Published:** 2023-09-11

**Authors:** Song-Jun Wang, Bing-Rui Liu, Fu Zhang, Xiao-Rui Su, Ya-Ping Li, Chen-Teng Yang, Zhi-Hua Zhang, Bin Cong

**Affiliations:** 1 College of Forensic Medicine, Hebei Medical University, Hebei Key Laboratory of Forensic Medicine, Collaborative Innovation Center of Forensic Medical Molecular Identification, No. 361 Zhongshan East Road, Chang’an District, Shijiazhuang, 050017 Hebei China; 2 Forensic Pathology Lab, Guangdong Public Security Department, Guangzhou, China; 3 Department of Science and Education, Hebei Chest Hospital, No. 372 Shengli North Street, Chang’an District, Shijiazhuang, 050041 Hebei China

**Keywords:** Metabolomics, Metabolomics

## Abstract

This study differentiates myocardial infarction (MI) and strangulation death (STR) from the perspective of amino acid metabolism. In this study, MI mice model via subcutaneous injection of isoproterenol and STR mice model by neck strangulation were constructed, and were randomly divided into control (CON), STR, mild MI (MMI), and severe MI (SMI) groups. The metabolomics profiles were obtained by liquid chromatography-mass spectrometry (LC–MS)-based untargeted metabolomics. Principal component analysis, partial least squares-discriminant analysis, volcano plots, and heatmap were used for discrepancy metabolomics analysis. Pathway enrichment analysis was performed and the expression of proteins related to metabolomics was detected using immunohistochemical and western blot methods. Differential metabolites and metabolite pathways were screened. In addition, we found the expression of PPM1K was significantly reduced in the MI group, but the expression of p-mTOR and p-S6K1 were significantly increased (all P < 0.05), especially in the SMI group (P < 0.01). The expression of Cyt-C was significantly increased in each group compared with the CON group, especially in the STR group (all P < 0.01), and the expression of AMPKα1 was significantly increased in the STR group (all P < 0.01). Our study for the first time revealed significant differences in amino acid metabolism between STR and MI.

## Introduction

Determination of the cause of death is an indispensable part of judicial evidence collection and plays an important role in forensic medicine practice^1^. Nowadays, the determination or diagnosis of cause of death is performed primarily at the histological level by examining morphological lesions or changes during autopsy and tissue section observation^2^. However, the identification of cause of death is difficult due to the pathophysiological processes involved in death are still poorly understood. For example, myocardial infarction in sudden cardiac death is the most common pattern of death in forensic medicine and is mostly caused by coronary atherosclerotic heart disease. But it is difficult to determine cause of death as myocardial infarction when atherosclerosis of the coronary artery is not serious and morphological changes are nonspecific^3^. In addition, mechanical asphyxia (e.g. strangulation death) leads to death by external violence disrupting respiratory function, and the current determination of the cause of death is based on the full findings from the autopsy and case analysis. However, the identification of cause of death as mechanical asphyxia always causes some errors because of lacking specific signs^4^. Moreover, in trials, forensic pathologists are often requested to distinguish between primitive cardiac causes myocardial infarction and strangulation death. Thus, selecting effective indicators for the identification of cause of death in myocardial infarction and strangulation death and elucidating the different pathophysiological processes are the focus of forensic medicine research.

Metabolomics refers to the analysis of metabolites in biological fluids or tissues, which focuses on discovering the biomarker with the aim of the identification of metabolites that is relevant to various diseases^5^. Metabolomics is a tool that is used to analyze the metabolic response of various diseases, identify potential diagnostic or prognostic biomarkers, and discover toxicity-related or drug-related metabolic pathways, which has attained good prospects^6–10^. More importantly, metabolomics has also been widely used in the field of forensic medicine^11^. Dian Wang et al*.* studied the metabolomics alterations associated with acute myocardial ischemia in the rat myocardium and identified several important metabolites that provide new clues in forensic medicine^12^. Dimitrios et al*.* found succinate overproduction in asphyxia conditions by analyzing the metabolomics alterations in animal plasma, suggesting succinate metabolism has a potential prognostic value^13^. Amino acids are the main metabolites for maintaining cell survival and play regulatory roles in key intracellular signaling pathways^14^. Meanwhile, amino acids, a hot spot in metabolomics research, are used as biomarkers to identify various diseases^15^. Previous studies have shown that the dysmetabolism of amino acids especially branched-chain amino acids (BCAAs) contributes to myocardial infarction cardiac dysfunction and remodeling^16^. Several metabolomics studies to determine biomarkers of cause of death in forensic practice have found that amino acids could be potential metabolites^2,13,17^. However, there are lacking evidence on whether amino acids can be used as a valid target to discriminate between myocardial infarction and strangulation death. Thus, we conducted experiments to elucidate the alterations of metabolites in myocardial infarction and strangulation death from the perspective of amino acid metabolism and its signaling pathways to find potential diagnostic indicators to differentiate the cause of death.

## Material and methods

### Experimental protocol

Forty-eight clean-grade healthy inbreed male C57 mice (9 weeks old, weight 20 ± 2 g) were obtained from the Experimental Animal Center of Beijing University of Medical Sciences (Beijing, China). All mice maintained under identical housing (housed under a 12 h light/dark cycle environment with a constant temperature of 23 ± 2 °C and relative humidity of 50%) and feeding conditions. All animals were adaptive to a new environment for 7 days before starting the experiment. Animal procedures were conducted according to the Experimental Animal Research Protocol approved by the Laboratory Animal Management Committee of Hebei Medical University (No.20223011), and all experiments were performed in accordance with relevant guidelines and regulations. Here we state that all methods in this study are reported in accordance with ARRIVE guidelines.

Forty-eight animals were randomly divided into 4 groups including control (CON, n = 12), STR (n = 12), mild MI (MMI, n = 12), and severe MI (SMI, n = 12) groups. The mice were anesthetized by intraperitoneal injection of 1% sodium pentobarbital. For the mice model of CON, the mice were subjected to decapitation without any treatment under anesthesia. For the STR group, strangulation was generated through ligature. A noose made of cotton thread was placed around the neck, and a small stick was inserted into the noose from the back of the neck. Then, the noose was tightened by rotating the stick to asphyxiate the rat under steady pressure until the rat died (approximately 4–5 min). For the MMI group, death was caused after 10 min intraperitoneal injection with 1 g/kg isoproterenol. For the SMI group, death was caused after 3 min intraperitoneal injection with 1.5 g/kg isoproterenol. The mice models in all groups were verified using hematoxylin–eosin (HE) staining. Left ventricular wall myocardial tissues were harvested to make sections for subsequent metabolomics analysis, liquid chromatography-mass spectrometry (LC–MS) assay of ceramide, immunohistochemistry, and western blot.

### Sample preparation

For metabolomics analyses, quickly removed the heart tissue of mice on ice and wiped off excess blood. The heart tissue was added hypothermic saline and homogenized. Then placed it into a freezing tube and quenched in liquid nitrogen for 15 min. A portion of the left ventricular wall tissue was extracted and stored frozen at – 80 °C for western blot analyses. In addition, a portion of the left ventricular wall tissue was extracted and fixed in 10% formaldehyde solution, dehydrated in gradient alcohol, soaked in paraffin, and a 5 μm-thick section was cut for histological examination.

The metabolomics profiles were obtained with an ACQUITY UPLC I-Class plus-QE plus combined system (Thermo Fisher Scientific, USA). Chromatographic separation was performed on an ACQUITY UPLC HSS T3 column (2.1 × 100 mm, 1.8 μm, America Advanced Material Technology Cor.) with a constant temperature of 35°C. The mobile phases were water with 0.1% formic acid as solvent A and acetonitrile with 0.1% formic acid as solvent B. The flow rate was 0.35 mL/min and the injection volume was 2 μL. The gradient elution procedure was as follows: 0–2 min, 5% B; 2–4 min, 5–30% B; 4–8 min, 30–50% B; 8–10 min, 50–80% B; 10–15 min, 80–100% B; 15–16 min, 100–5%. The positive and negative ion scanning modes were processed to collect the quality spectrum signal of the samples, which were equipped with an electrospray ionization (ESI) source.

### Data analysis of metabonomics

Before data pre-processing for pattern recognition, raw data were processed by metabolomics software Progenesis QI v2.3 software (Nonlinear Dynamics, Newcastle, UK) for baseline filtering, peak identification, integration, retention time correction, peak alignment, and normalization. The precursor tolerance was set at 5 ppm, product tolerance was set at 10 ppm, and the product ion threshold was set at 5%. Compound identification was based on precise mass numbers, secondary fragmentation, and isotopic distribution according to the Human Metabolome Database (HMDB), Lipidmaps (v2.3), and METLIN databases as well as self-built libraries for characterization. For the extracted data, the ion peaks with the missing value > 50% were deleted and the 0 values were replaced by half of the minimum values. The compounds obtained from the characterization were screened according to a score of 36 (the total score was 60), and the results below 36 were considered inaccurate and deleted. Then, the positive and negative ion data were combined into a data matrix that contains all the information extracted from the raw data that could be used for analysis, and as the basis for subsequent analysis.

Principal component analysis (PCA) merging method with unsupervised was used to recognize the distribution status of the overall sample, natural aggregation, and abnormal samples. The aggregation of the quality control (QC) sample was used to evaluate the quality of data and QC samples were prepared by mixing the extracts of all samples in equal volume (a QC sample was injected every 10 samples during the whole analysis). The supervised orthogonal partial least squares-discriminant analysis (OPLS-DA) model and partial least squares-discriminant analysis (PLS-DA) were applied to maximize inter-group discrimination. Before multivariate analysis, the data was log-transformed and normalized by using Pareto scaling. R2x (for interpretation ability) and Q2y (for prediction ability) provided a measure of the OPLS-DA model fit. The 200 permutation testing were applied to check the risk of overfitting in OPLS-DA models. The most important metabolites were selected by variable importance in projection (VIP) value that was obtained based on OPLS-DA. The fold change analysis (FC) and Wilcoxon test were conducted between the control and experimental group to discover metabolic characteristics. In addition, a t-test was further used to verify the significance of differential metabolites between groups. In the OPLS-DA model, the metabolites with variable important in projection (VIP) > 1.0, FCs > 1.5 or < 0.6667 (the FC cutoff was 1.5), and p values of t-test < 0.05 were considered statistically significant^18^.

PCA plots, S-plots, volcano plots, and heatmaps were constructed and metabolites with VIP values > 1.0 and p values of t-test < 0.05 were labeled. Metabolites that increase or decrease were marked red or blue, respectively. The pathway enrichment analysis was performed by the KEGG database.

### Immunohistochemical analyses

Anti-AMPKα1 (ab32047), Cyt-C (ab133504), mTOR (ab109268), PPM1K (ab135286), and S6K1 (ab32529) (Abcam, Cambridge, UK) antibodies were applied in 1:100 dilutions at 4°C for 12 h according to the protocol of the immunohistochemistry kit. Left ventricular wall myocardial tissues were incubated with biotin-labeled secondary antibody for 1 h, followed by horseradish peroxidase for 30 min. 3,3'-Diaminobenzidine (DAB) was used as the chromogenic agent to detect the target protein. Hematoxylin was used as a nuclear re-dyeing agent. Exclude negative controls for each tissue section of the primary antibody and perform appropriate positive controls for each set of sections. Experimental conditions remain stable throughout the process. Slides for immunohistochemistry were scanned using an Aperio ScanScope (Aperio Technologies, Vista, CA, USA). After saving each digital image, the left ventricular wall region (excluding edge effects) was selected for analysis. The average positive intensity of AMPKα1, Cyt-C, mTOR, PPM1K, and S6K1 positive cells was assessed using computer-aided image analysis and the cytoplasmic V2.0 algorithm of the Aperiosmescope software (Aperio, Vista).

### Western blot analyses

Protein extracts were obtained from the left ventricular wall myocardial with a mammalian protein extraction reagent (Pierce, ThermoScientific, Rockford, IL, USA). Protein concentration was determined using a bicinchoninic acid protein assay. The protein extracts of 50 µg were separated by SDS-PAGE under reducing conditions and electrophoretically transferred onto a polyvinylidene fluoride membrane. Proteins of interest were detected with specific antibodies: anti-AMPKα1, anti- Cyt-C, anti-mTOR, anti-PPM1K, and anti-S6K1 at 1:1000 dilution each. Then incubated with a re-probed with horseradish peroxidase-labeled secondary antibody coupled with labeled chemiluminescence or fluorescent molecule, visualized all band intensities. Thereafter, the protein was detected using SuperSignal substrate and the quantitative analysis was employed by the Image J software.

### Statistical analysis

The Kolmogorov–Smirnov test showed a normal distribution between all groups (*P* > 0.1) and data were presented as the mean ± standard error of the mean (SEM). The significance of the differences between the two groups was determined by t-tests. The statistical differences among three or more groups were evaluated by one-way ANOVA with tukey’s post hoc test. There was a statistically significant difference between the two groups when the p-value < 0.05.

### Ethics statement

The animal use protocol for this study has been reviewed and approved by the Institutional Review Board for Animal Experiments at Hebei Medical University, and all experiments were performed in accordance with relevant guidelines and regulations. Meanwhile, all methods in this study are reported in accordance with ARRIVE guidelines.

## Results

### Successful construction of mice models

We evaluated the success of the construction of the mice model by HE staining results. The results of HE staining (Supplementary Fig. S1) showed deeper and enhanced in the experimental group than control group, which might be ascribed to the increased eosinophilic components. It might indicate the occurrence of heart injury. Among them, cardiomyocytes showed excessive contraction leading to rupture and eosinophilic variation of endochylema in STR group; cardiomyocytes contained dispersed lamellar in MMI group; cardiomyocytes contained lamellar in SMI group. The results showed that cardiomyocyte injury and energy metabolism disorder occurred in the experimental group compared to the control group. These findings suggested that well-established mice models in the experimental groups were different from those in the control group.

### PCA, PLS-DA, and OPLS-DA analysis

**Figure 1:**
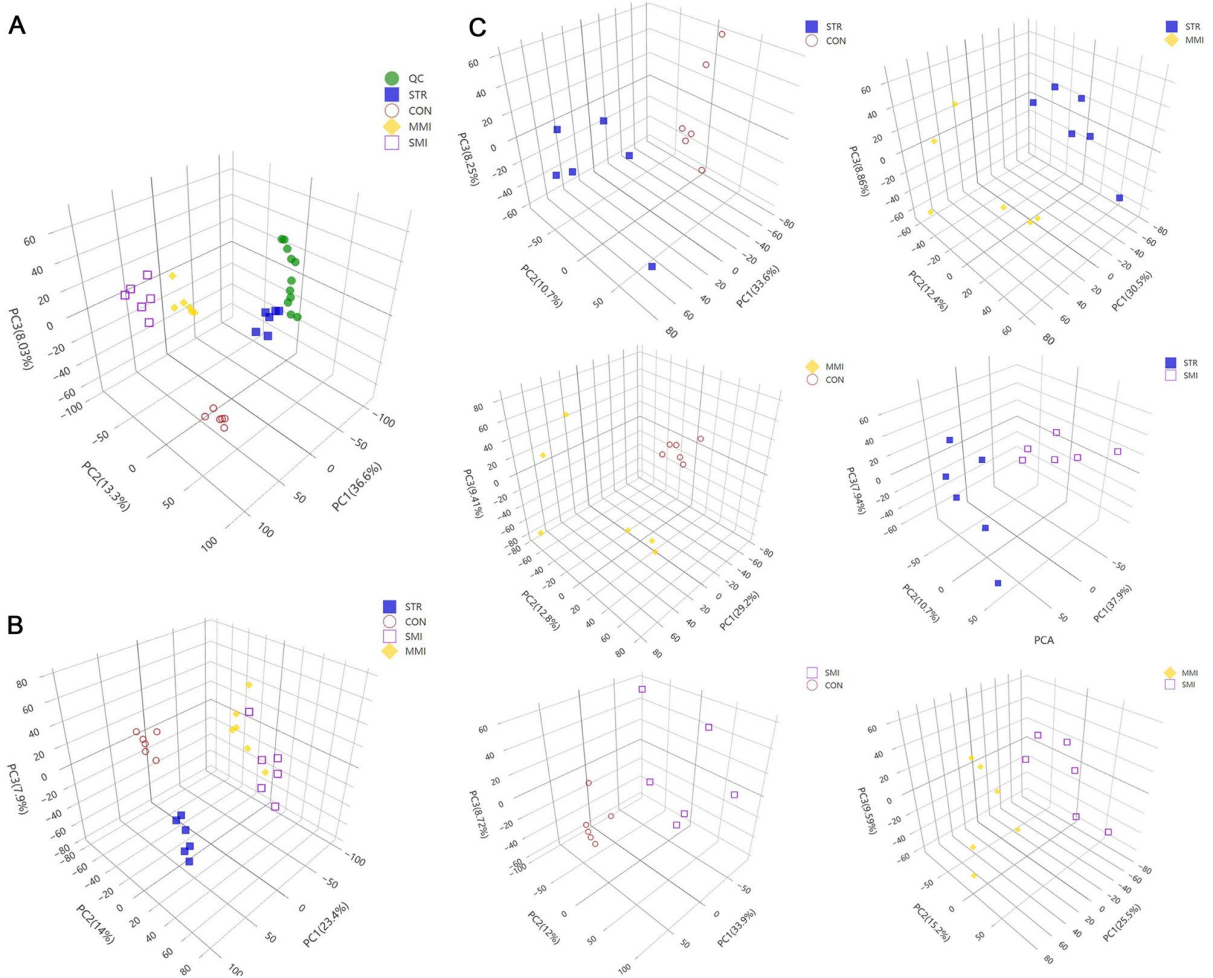
PCA scores plots. (**A**) The PCA score plot of all groups; (**B**) The PCA score plot of the STR group, MMI group, and SMI group. (**C**) The PCA score plot of the CON vs. STR group, CON vs. MMI group, CON vs. SMI group, STR vs. MMI group, STR vs. SMI group, and MMI vs. SMI group.

After preprocessing the original data of left ventricular wall tissue samples, PCA was performed to separate the samples and visualize the overall differences in the data set. The result of PCA score plots showed that all QC samples clustered together and did not show a separation trend, indicating the collection and analysis of the data were performed as reliable, stable, and correct (Fig. 1A). The PCA completely separated into different groups, and each group showed definite differences in distribution compared to the other groups, suggesting that the distribution of metabolites differed across the samples of each control group (Fig. 1B). Moreover, PCA analysis between each of the two groups further confirmed that each sample was classified into different metabolite profiles (CON vs. STR, CON vs. MMI, CON vs. SMI, STR vs. MMI, STR vs. SMI, and MMI vs. SMI, Fig. 1C), indicating considerable differences in metabolite profiles in samples across each cause of death and the control group, and across each control group.

**Figure 2:**
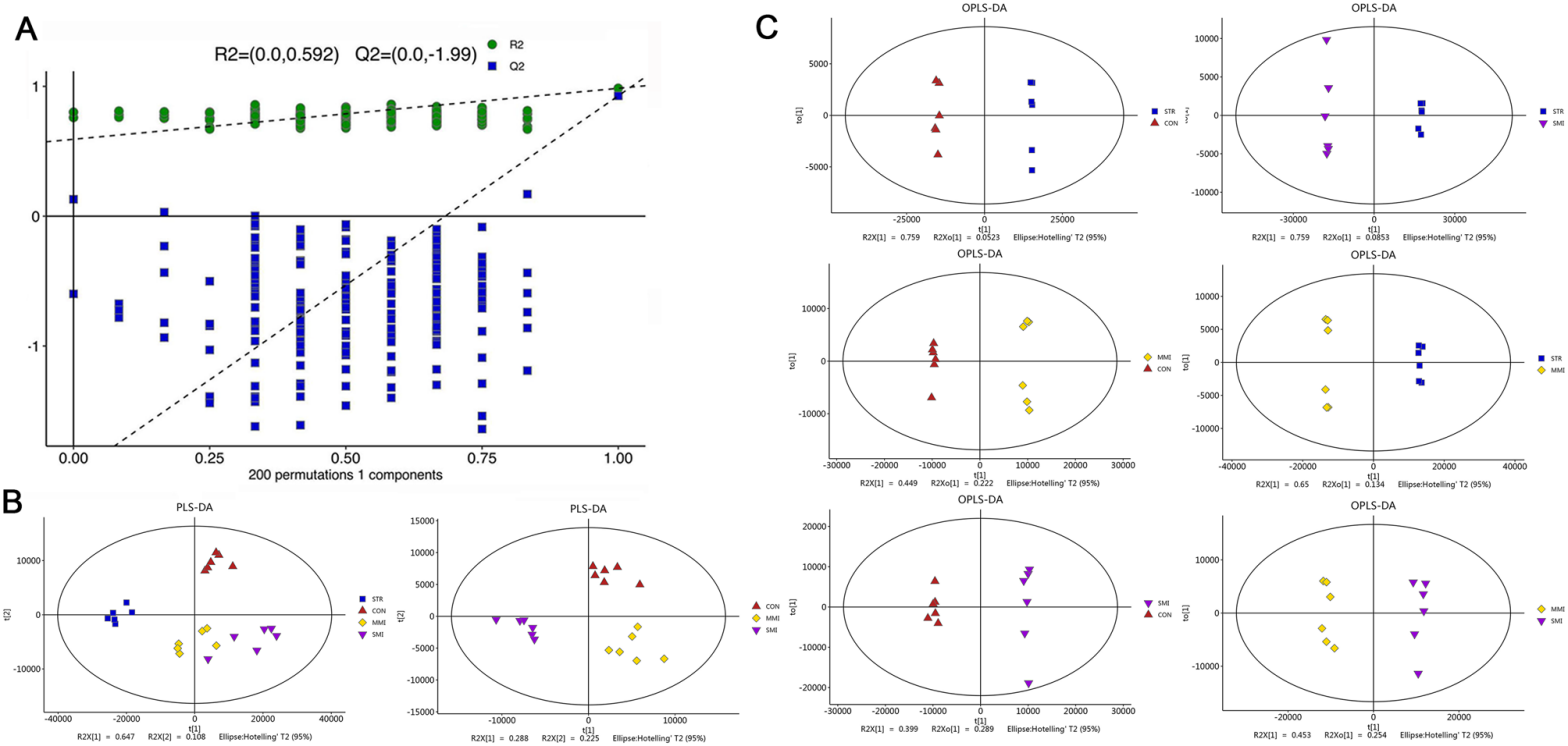
PLS-DA and OPLS-DA analysis. (**A**) The permutation test (200 times) of the OPLS-DA model; (**B**) PLS-DA score plot of all groups and MI vs. CON groups; (**C**) OPLS-DA score plot of CON vs. STR group, CON vs. MMI group, CON vs. SMI group, STR vs. MMI group, STR vs. SMI group, and MMI vs. SMI group.

In order to further analyze the distribution of metabolites in STR and MI samples, the orthogonal partial least square discrimination analysis (OPLS-DA) model was built. Firstly, the cross-validation with 200 permutation tests showed that this OPLS-DA model was reliable, with intercepts of R^2^ and Q^2^ equal to 0. 592 and − 1.99, respectively (Fig. 2A). The R^2^ and Q^2^ values of the OPLS-DA model between the two groups were greater than 0.5 and close to 1, which revealed the separation was effective. Next, we established two PLS-DA (all groups and CON vs. MI) and six OPLS-DA models (CON vs. STR, CON vs. MMI, CON vs. SMI, STR vs. MMI, STR vs. SMI, and MMI vs. SMI). The PLS-DA score plots showed a better separation for all groups, suggesting different significant metabolic profiles between groups; MMI group were closer to the SMI group, suggesting that the metabolic composition of the two groups was more similar (Fig. 2B). The OPLS-DA score plots showed there was a clear trend of clustering and were well differentiated between groups. Among them, STR clusters together tighter with a greater intergroup variability than MMI and SMI, even when compared to controls. This may suggest that STR had a stronger effect on metabolism (Fig. 2C).

### Screening and classification analysis of differential metabolites

**Figure 3:**
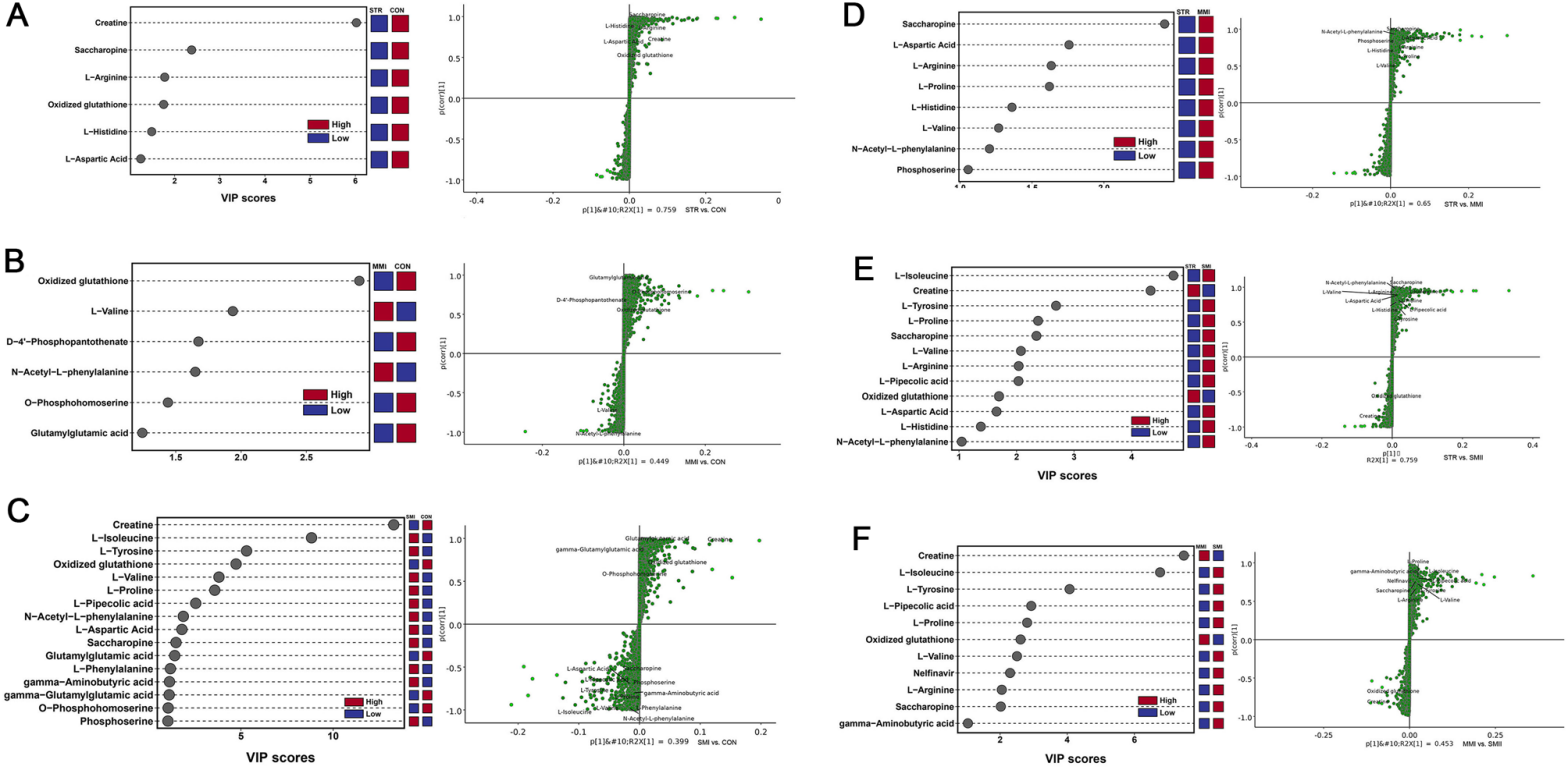
VIP plots and S-plots. (**A**) VIP plot and S-plot of CON vs. STR group; (**B**) VIP plot and S-plot of CON vs. MMI group; (**C**) VIP plot and S-plot of CON vs. SMI group; (**D**) VIP plot and S-plot of STR vs. MMI group; (**E**) VIP plot and S-plot of STR vs. SMI group; F: VIP plot and S-plot of MMI vs. SMI group. VIP plot with VIP value > 1. In S-plot plot, the variables far from the origin contributed significantly to differentiate the clustering between two groups and were considered as potential biomarkers.

In the OPLS-DA model, VIP scores of metabolites were used to confirm the metabolites contributing to separate groups. The metabolites were selected as potential biomarkers based on the VIP of the results of PLS-DA (VIP > 1)^19^, fold-change threshold (FC ≥ 1.5 or FC ≤ 0.5), and t-test threshold (*P* < 0.05). We used the S-plot and VIP plots to visualize the influences of variables when selecting metabolites with strong model contributions and high statistical reliability. The results showed that, compared with the control group, 6, 6, and 16, respectively metabolites were responsible for the discrimination of metabolites in the STR group, MMI group, and SMI group (all *P* < 0.05; Fig. 3A–C). Compared with the STR group, there were 8 and 12, respectively differential metabolites in the MMI group, and SMI group (all *P* < 0.05; Fig. 3D,E). There were 11 metabolites characterized by the metabolic discriminations between the MMI group and SMI group (all *P* < 0.05; Fig. 3F). The differential metabolites related to the three cause of death groups were detailed in Supplementary Table S1.

**Figure 4:**
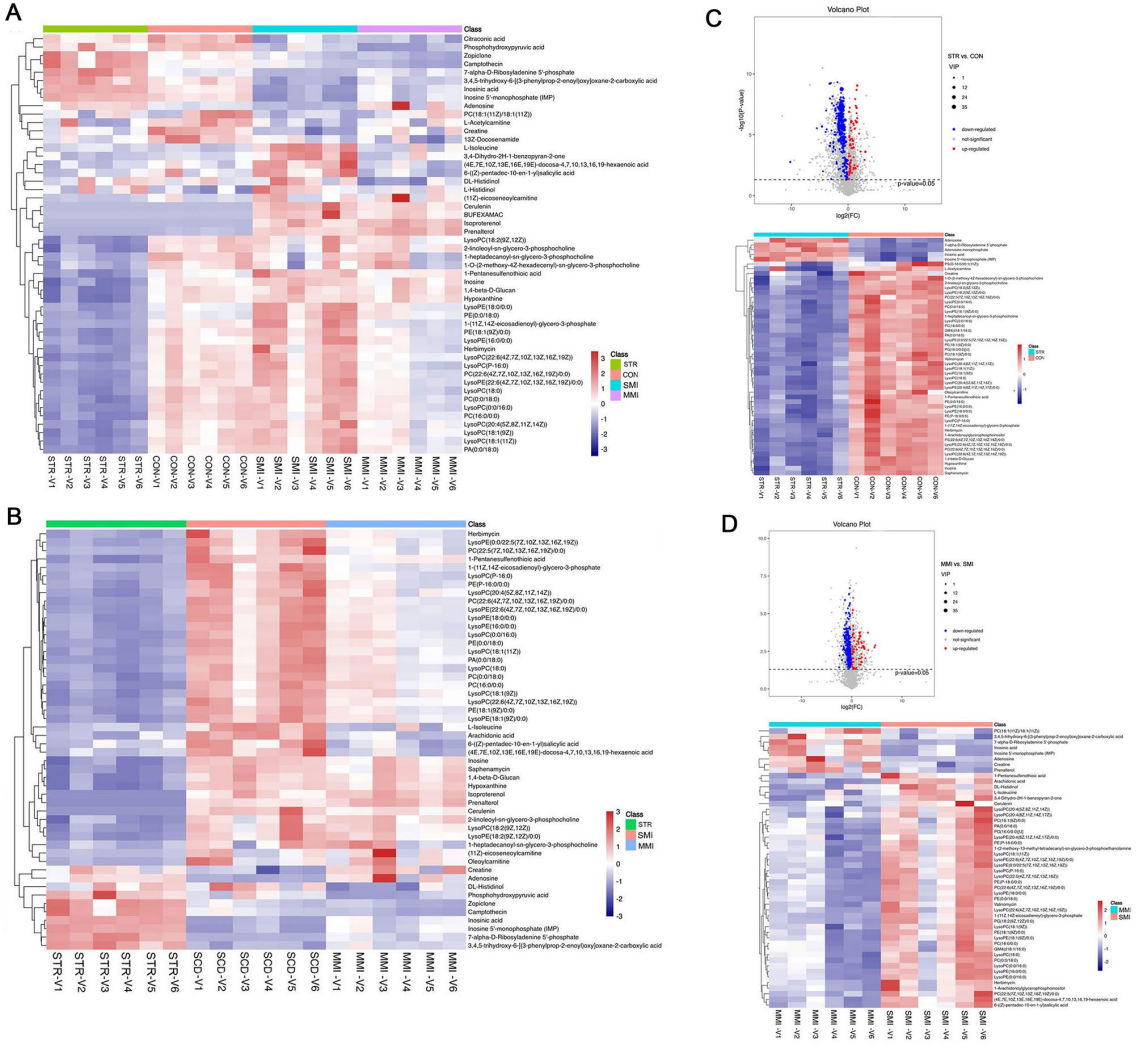
Volcano plot and heatmap. (**A**) Heatmap of top 50 metabolites of all groups; (**B**) Heatmap of top 50 metabolites of STR group, MMI group, and SMI group; (**C**) volcano plot and heatmap of top 50 metabolites in CON vs. STR group; (**D**) Volcano plot and heatmap of top 50 metabolites in MMI vs. SMI group. In volcano plot, the red dots represent up-regulated, the blue dots represent down-regulated, and the grey dots represent no significant difference (namely, metabolites that are detected but did not meet the filtering parameters for screening). R language “limma” package (http://www.bioconductor.org/packages/release/bioc/html/limma.html) was applied to identify diferentially expressed genes.

A volcano plot containing the p values of the student’s t-test and fold changes was performed among different groups to identify the discrepancy in metabolite distribution. The heatmap depicted the expression chances of the top 50 discrepancy metabolites by the most significant differences (VIP). The result of the heatmap showed again the obvious discrepancy in amino acid metabolism between the different cause of death (all *P* < 0.05; Fig. 4A). The result of STR vs. MI (all *P* < 0.05; MMI group and SMI group) group showed inosine, arachidonic acid, L-isoleucine, hosphohydroxypyruvic acid were decreased in the STR group (all *P* < 0.05; Fig. 4B). Compared with the control group, creatine and hypoxanthin were decreased, but adenosine and inosinic acid were increased in the STR group (all *P* < 0.05; Fig. 4C). Moreover, creatine was increased and L-Isoleucine was decreased in the SMI group when compared with the MMI group (all *P* < 0.05; Fig. 4D). Other groups (all *P* < 0.05; CON vs. MMI, CON vs. SMI, STR vs. MMI, and STR vs. SMI; Supplementary Fig. S2) of volcano plots and heatmaps also show the obvious discrepancy.

### Enrichment analysis of metabolomics pathway

**Figure 5:**
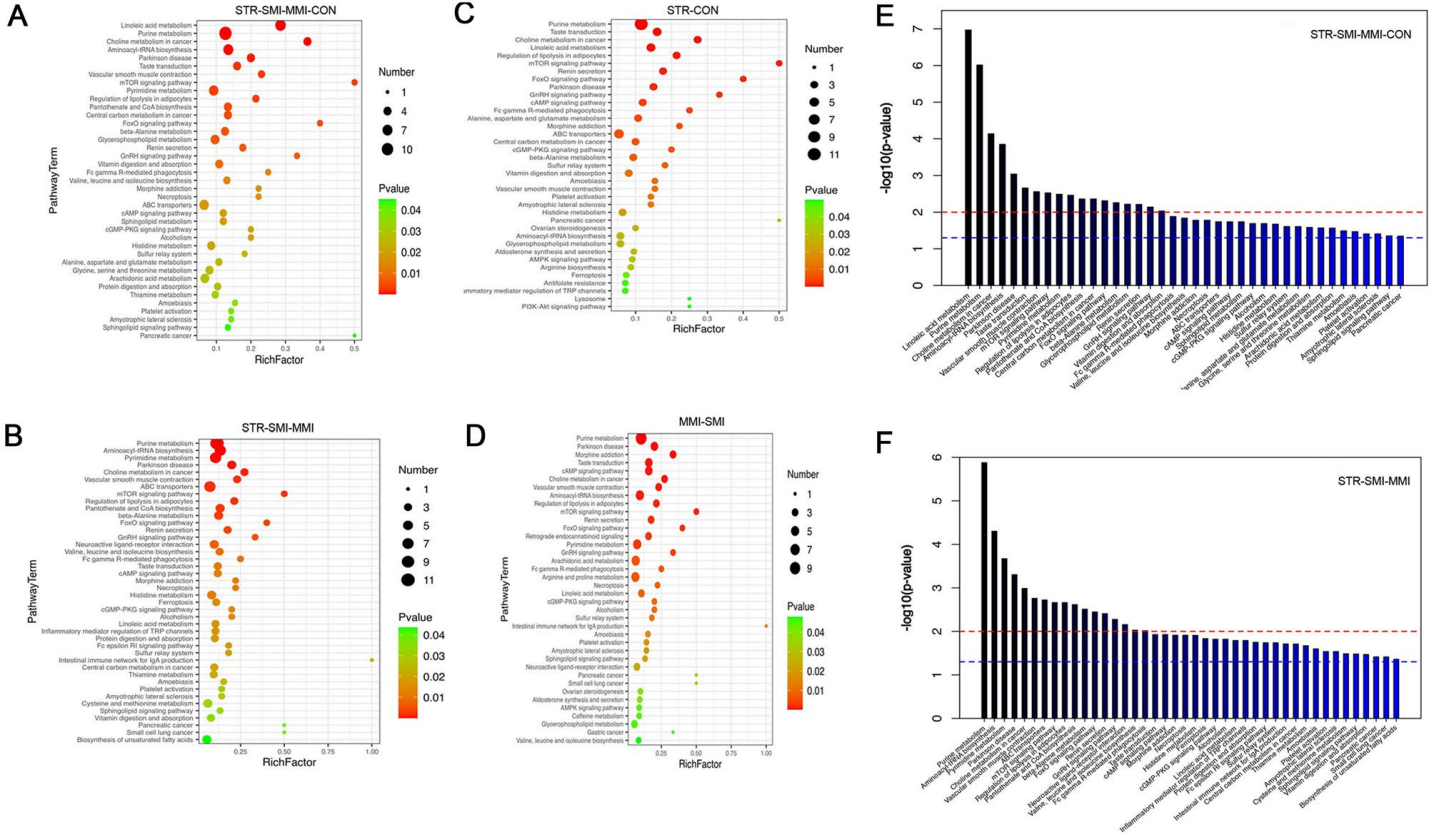
Bubble chart and bar chart of KEGG enrichment analysis. (**A**) Bubble chart of KEGG enrichment in all groups. (**B**) Bubble chart of KEGG enrichment among STR group, MMI group, and SMI group. (**C**) Bubble chart of KEGG enrichment in CON vs. STR group; (**D**) Bubble chart of KEGG enrichment in MMI vs. SMI group. (**E**) Bar chart of KEGG enrichment in all groups; (**F**) bar chart of KEGG enrichment among STR group, MMI group, and SMI group.

To further investigate the metabolomics pathways between the different cause of death, we tried to use bubble charts to show the possible related metabolomics pathways. The result showed that the metabolomics were mainly enriched in the following metabolomics pathways: lincieic acid, aminoacyl-tRNA biosynthesis, mTOR signaling, and purine (all *P* < 0.05; Fig. 5A). Compared with the MI group, the significantly different metabolomics signaling pathways in the STR group were mainly enriched in aminoacyl-tRNA biosynthesis, mTOR signaling, and beta-alanine metabolism (all *P* < 0.05; Fig. 5B). The enrichment analysis of group STR vs. CON showed that ampk signaling pathway was also enriched (*P* < 0.05; Fig. 5C). Moreover, when comparing the SMI group with the MMI group, pathways including purine, aminoacyl-tRNA biosynthesis, and mTOR signaling were enriched (all *P* < 0.05; Fig. 5D). The result of bubble charts in other groups (CON vs. MMI, CON vs. SMI, STR vs. MMI, and STR vs. SMI;) showed in Supplementary Fig. S3. In addition, the results of metabolic pathway classification suggested that a large proportion of metabolism was enriched in multiple pathways associated with amino acid metabolisms such as aminoacyl-tRNA biosynthesis, beta-alanine metabolism, and mTOR signaling (all *P* < 0.05; Fig. 5E). This enrichment result was similar to the enrichment analysis of STR and MI groups (all *P* < 0.05; Fig. 5F).

### Immunohistochemical and western blot analyses

**Figure 6:**
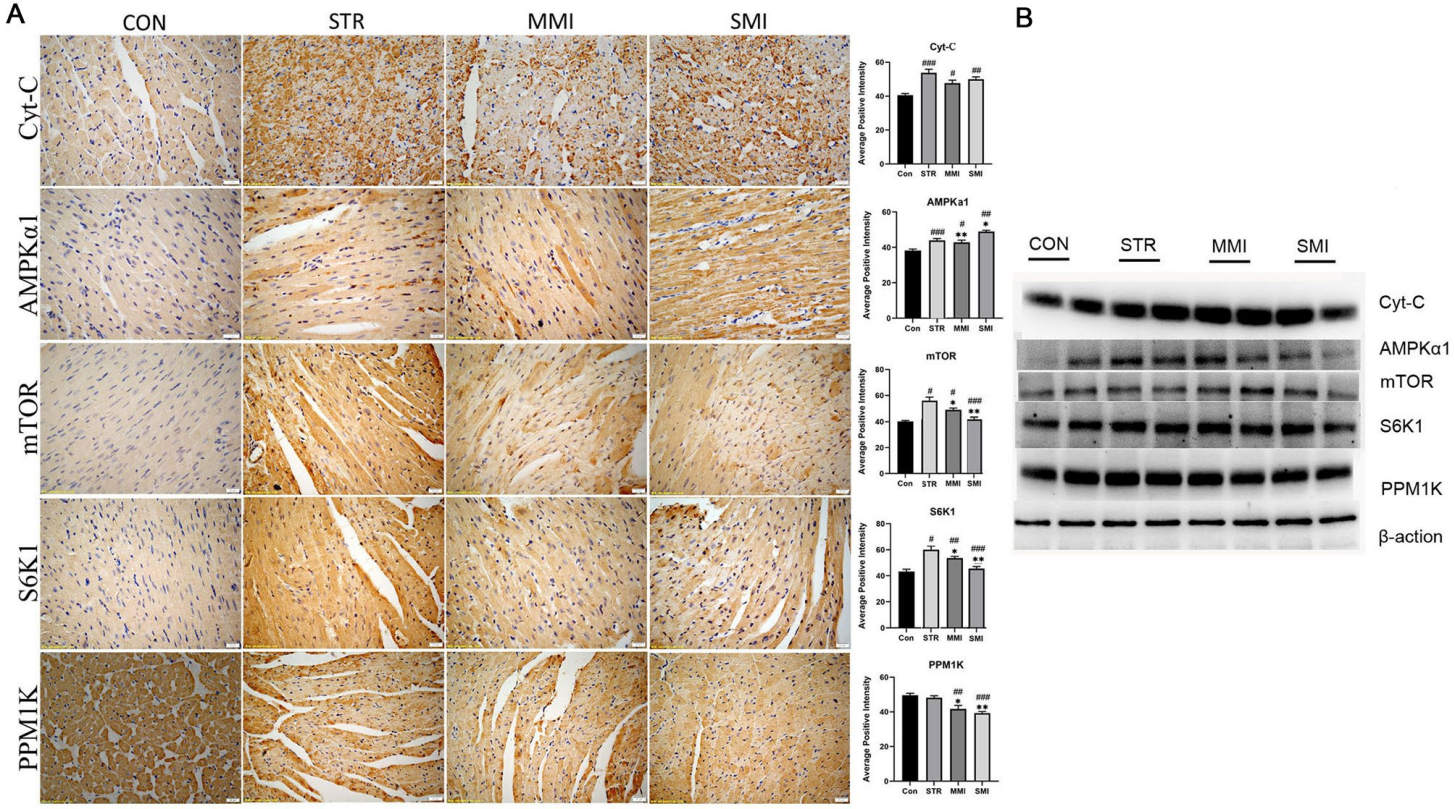
Immunohistochemical and western blot analyses of the level of expression of proteins related to amino acid metabolic events. (**A**) The expression of AMPKα1, Cyt-C, mTOR, S6K1, and PPM1K by immunohistochemical (40 × , bar = 500 μm). (**B**) The expression of AMPKα1, Cyt-C, mTOR, S6K1, and PPM1K by western blot. (*Compared with STR group, p < 0.05; **p < 0.01; ^#^Compared with CON group, p < 0.05; ^##^p < 0.01.). Full-length blots, used to create this (**B**), are available in the Supplemental Fig. S4.

To assess the level of expression of proteins related to amino acid metabolomics events in the different cause of death processes, immunohistochemical and western blot were performed. As the result shown in Fig. 6A, the expression of Cyt-C was significantly increased in each experimental group compared with the CON group, especially in the STR group (all *P* < 0.01). The expression of AMPKα1, mTOR, and S6K1 was significantly increased (all *P* < 0.05), while PPM1K was reduced in the MI group and STR group when compared with CON groups. Moreover, compared with the STR group, the expression of AMPKα1 was significantly increased while mTOR and S6K1 were significantly reduced in the MI group (all *P* < 0.05), especially the change was more significant in the SMI group (*P* < 0.01). Similar results were also observed by the western blot (Fig. 6B).

## Discussion

Identifying cause of death is often the primary goal of the practice of forensic medicine, especially in cases of unexpected sudden death usually caused by heart disease^20^. The main method of determining the cause of death is based on macroscopic and microscopic morphological features^2^. However, myocardial infarction, a common cause of sudden cardiac death, is often difficult to distinguish from asphyxial death (such as strangulation death) when corpse signs may be nonspecific, unrepresentative, or even absent, or when the deceased has an underlying disease that can lead to sudden unexpected death^21,22^. The existing studies suggest that amino acid metabolism may play an important role in myocardial infarction and mechanical asphyxia^23,24^, but researches that have focused on identifying the cause of death in myocardial infarction and asphyxial death are limited. Therefore, investigating the amino acid metabolomics features and identifying candidate biomarkers in different cause of death is essential.

First, we preliminary revealed the expression of metabolites in different cause of death by metabolomics. We visualized the differential expression of metabolites in each cause of death group by cluster analysis such as S-plot, heatmap, and volcano map, and we found metabolism varied significantly in different groups. This is consistent with a study by Zhang et al*.,* which found obvious changes in metabolite during different death processes by using untargeted metabolomics^2^. Meanwhile, OPLS-DA analysis of metabolite differences showed a greater intergroup variability in STR group than MI group in our study, suggesting there might be a stronger effect of asphyxial mechanism on metabolome. Two studies showed significant metabolic differences between ventricular fibrillation cardiac arrest and asphyxia cardiac arrest, as well as a a more significant correlation from asphyxia mechanism to metabolomics, which is consistent with our study^13,24^. Further, we have identified several different metabolites associated with amino acids altered in this study, which is consistent with a metabolomics study conducted by Yang et al*.* on rats with hypoxia and high-altitude pulmonary hypertension found significant changes in amino acid metabolism^25^. A study showed that hypoxia is associated with reduced l-arginine transport in normal cells^26^, which can explain our findings that L-arginine metabolism was downregulated in the STR group due to a sudden decrease of oxygen in asphyxial death. Moreover, proline is likely to provide an important energy source for organismal activity and can be converted to enter the metabolomics cycles such as the TCA cycle and yield Acetyl CoA^27^. In our study, we found that compared with the CON group, the expression of proline was significantly reduced in the STR group, suggesting that mechanical asphyxia caused by strangulation caused a significant increase in myocardial energy expenditure and proline decreased due to excessive depletion. However, BCAA such as L-valine and L-isoleucine was significantly increased in the MI group. This may be related to myocardial damage directly caused by the cardiotoxic effects of isoprenaline injection (a model of MI established by injecting isoproterenol in this experiment), which affects the metabolism of amino acids in the myocardium and lead to the accumulation of BCAAs. This is consistent with previous findings, Li et al*.* found amino metabolism dysfunction indirectly affects cardiac energy metabolism and Wahid et al*.* reported the accumulation of BCAAs in the heart during isoprenaline to induce myocardial resulted in a decrease in the levels of BCAAs in serum^28,29^.

Subsequently, we conducted the pathway analysis and explored potential biomarkers for differential metabolites. In our study, we identified 4 significantly different signaling pathways related to amino acid metabolism in the different groups, including aminoacyl-tRNA biosynthesis, beta-Alanine metabolism, the mTOR signaling pathway, and the AMPK signaling pathway. This is consistent with the findings in several studies on the pathway in mechanical asphyxia and myocardial infarction^30–32^. Creatine is a key regulator of energy metabolism and phosphocreatine is essential to maintain ATP levels in tissues with high energy demands^33^. Our study discovered that creatine and were increased in STR compared to SMI, suggesting in creatine levels were decreased and more severe energy impairment in the SMI group. When energy metabolism was impaired AMPK can be phosphorylation and then phosphorylated AMPK/AMPKα1 can reduce the activity of mTOR and S6K1^34,35^. This is consistent with our results of western blot and immunohistochemical, in our study compared with STR group, the expression leavels of AMPKα1 was significantly increased, and mTOR and S6K1 was significantly desearsed in the SMI gruop. In addition, BCAAs are essential amino acids and the accumulation of BCAAs have showed associated with many metabolomics diseases including MI^36,37^. Meanwhile, BCAAs metabolism levels were strongly associated with the PPM1K gene^38^. In our study, we found a significant decrease in the expression of PPM1K in the MI group, especially in the SMI group. This is also confirmed in another study by Peng et al*.* that the decrease in PPM1K led to the accumulation of the cellular BCAA, which affected myocardial function^39^. Moreover, BCAA accumulation can stimulate mTOR which regulates mTOR/S6K1 signaling and mediates various biological effects of insulin, and energy^38,40^. A recent report showed that amino acids affect insulin signaling through mTOR/S6K1 phosphorylation of IRS1^41^. In our study, we found that the expression of mTOR and S6K1 was significantly increased in the MI group, especially in the SMI group, suggesting that the disturbance of amino acid metabolism in myocardial infarction further triggered insulin resistance, leading to impaired energy metabolism in cardiac myocytes. This is consistent with a previous study showing amino acid induces cardiac insulin resistance to aggravate myocardial infarction^42^. In addition, Cyt-c is a small soluble electron carrier hemeprotein located in large amounts in the inner mitochondrial membrane, and leaks from the mitochondria to the cytoplasm when the impairment of the mitochondrial membrane potential^43^. Our study found that the expression of Cyt-C was significantly increased in experimental group especially in STR group, indicating that Cyt-C may be a valid biomarker for differentiating the cause of death. This is in agreement with previous study which have shown that Cyt-C increased significantly after cardiac arrest^44^.

However, this study has some potential limitations. First, these findings were drawn from the animal model, which was not enough to translate into the complex framework in humans. Second, targeted metabolomics was not conducted in our study, which could further validate the different metabolites identified by untargeted metabolomics. Third, the experiments performed on biomarkers of different cause of death in this study are still relatively limited and further verification is needed to identify the precise biomarkers and their functions.

## Conclusion

Our study revealed significant differences in amino acid metabolism between strangulation death and myocardial infarction and due to different causes and mechanism of death. In parallel, we found some amino acid metabolism, especially BBCA metabolism, and its signaling proteins may be effective biomarkers to discriminate between strangulation death and myocardial infarction. This study provides a practicable path to study the determination of complex causes of death. Besides, the identification of specific profiles or potential biomarkers of amino acids may help in building causative hypothesis to be tested. In the future, this could be investigated in human beings by using quantitative detection and confirmatory experiments. Meanwhile, further studies are needed to explore its precise biomarker and its mechanism to promote the determination of cause of death in the future.

### Supplementary Information

Supplementary Information.

## Data Availability

The datasets analyzed during the current study are available from the corresponding author upon reasonable request.
