# Supplementary material for: The amino acid metabolomics signature of differentiating myocardial infarction from strangulation death in mice models
Source: Sci Rep. 2023 Sep 11;13:14999. doi: 10.1038/s41598-023-41819-6 (PMC10495377; doi:10.1038/s41598-023-41819-6)
Supplement: Supplementary file 1 — Supplementary Information. [file 41598_2023_41819_MOESM1_ESM.docx]

**Supplementary Table 1** The results of differential metabolites among three groups of causes of death

|  | Metabolites | Chemical class | RT (min) | P value | Log2FC | VIP | Tend | CV (%) |
| --- | --- | --- | --- | --- | --- | --- | --- | --- |
| STR vs. CON | Creatine | Amino acids | -0.11 | 2.10E-07 | -1.66 | 1.500 | Down | 2.88E+00 |
|  | L-Arginine | Amino acids | -0.52 | 3.51E-05 | -1.65 | 2.640 | Down | 7.70E+00 |
|  | L-Aspartic Acid | Amino acids | -0.58 | 2.30E-03 | -0.87 | 2.876 | Down | 6.23E+00 |
|  | L-Histidine | Amino acids | -0.49 | 1.18E-02 | -0.22 | 2.335 | Down | 7.60E+00 |
|  | Oxidized glutathione | Amino acids | -0.11 | 3.94E-07 | -1.51 | 1.588 | Down | 5.63E+00 |
|  | Saccharopine | Amino acids | -1.74 | 2.70E-02 | -0.35 | 5.139 | Down | 8.29E+00 |
| SMI vs. CON | Creatine | Amino acids | 0.72 | 1.26E-05 | -0.19 | 13.284 | Down | 2.88E+00 |
|  | gamma-Aminobutyric acid | Amino acids | 0.70 | 1.74E-03 | 0.88 | 1.132 | Up | 4.07E+00 |
|  | L-Aspartic Acid | Amino acids | 0.70 | 9.52E-03 | 0.40 | 1.805 | Up | 6.23E+00 |
|  | L-Isoleucine | Amino acids | 1.41 | 5.85E-06 | 0.68 | 8.830 | Up | 2.76E+00 |
|  | L-Phenylalanine | Amino acids | 2.19 | 1.03E-04 | 0.75 | 1.181 | Up | 6.26E+00 |
|  | L-Pipecolic acid | Amino acids | 0.63 | 1.53E-02 | 0.28 | 2.554 | Up | 3.30E+00 |
|  | L-Proline | Amino acids | 0.75 | 1.18E-04 | 0.52 | 3.584 | Up | 3.09E+00 |
|  | L-Tyrosine | Amino acids | 1.13 | 8.77E-04 | 0.57 | 5.308 | Up | 3.94E+00 |
|  | L-Valine | Amino acids | 0.81 | 9.97E-05 | 0.67 | 3.811 | Up | 4.43E+00 |
|  | N-Acetyl-L-phenylalanine | Amino acids | 4.96 | 3.04E-08 | 4.25 | 1.880 | Up | 7.56E+00 |
|  | O-Phosphohomoserine | Amino acids | 0.70 | 1.98E-02 | -0.32 | 1.060 | Down | 4.45E+00 |
|  | Oxidized glutathione | Amino acids | 1.06 | 2.11E-03 | -0.26 | 4.736 | Down | 5.63E+00 |
|  | Phosphoserine | Amino acids | 0.70 | 4.27E-03 | 0.54 | 1.046 | Up | 8.17E+00 |
|  | Saccharopine | Amino acids | 12.18 | 4.98E-02 | 0.26 | 1.486 | Up | 8.29E+00 |
|  | Glutamylglutamic acid | Amino acids | 0.77 | 1.58E-06 | -1.57 | 1.417 | Down | 2.66E+00 |
|  | gamma-Glutamylglutamic acid | Amino acids | 0.77 | 1.60E-03 | -1.79 | 1.115 | Down | 4.72E+00 |
| MMI vs. CON | D-4'-Phosphopantothenate | Amino acids | 1.25 | 9.60E-03 | -0.25 | 1.674 | Down | 4.24E+00 |
|  | L-Valine | Amino acids | 0.81 | 3.33E-02 | 0.27 | 1.937 | Up | 4.43E+00 |
|  | N-Acetyl-L-phenylalanine | Amino acids | 4.96 | 9.73E-06 | 3.94 | 1.650 | Up | 7.56E+00 |
|  | O-Phosphohomoserine | Amino acids | 0.70 | 6.76E-04 | -0.50 | 1.440 | Down | 4.45E+00 |
|  | Oxidized glutathione | Amino acids | 1.06 | 2.97E-02 | -0.12 | 2.905 | Down | 5.63E+00 |
|  | Glutamylglutamic acid | Amino acids | 0.77 | 3.64E-05 | -1.07 | 1.244 | Down | 4.72E+00 |
| STR vs. SMI | Creatine | Amino acids | 0.72 | 3.91E-03 | 0.08 | 4.330 | Up | 2.88E+00 |
|  | L-Arginine | Amino acids | 0.65 | 3.78E-05 | -0.74 | 2.037 | Down | 7.70E+00 |
|  | L-Aspartic Acid | Amino acids | 0.70 | 1.59E-04 | -0.99 | 1.652 | Down | 6.23E+00 |
|  | L-Histidine | Amino acids | 0.63 | 2.15E-03 | -0.57 | 1.380 | Down | 7.60E+00 |
|  | L-Isoleucine | Amino acids | 1.41 | 1.30E-05 | -0.62 | 4.726 | Down | 2.76E+00 |
|  | L-Pipecolic acid | Amino acids | 0.63 | 1.14E-03 | -0.50 | 2.034 | Down | 3.30E+00 |
|  | L-Proline | Amino acids | 0.75 | 6.50E-05 | -0.78 | 2.374 | Down | 3.09E+00 |
|  | L-Tyrosine | Amino acids | 1.13 | 1.30E-02 | -0.56 | 2.687 | Down | 3.94E+00 |
|  | L-Valine | Amino acids | 0.81 | 1.05E-04 | -0.63 | 2.076 | Down | 4.43E+00 |
|  | N-Acetyl-L-phenylalanine | Amino acids | 4.96 | 3.63E-08 | -3.90 | 1.048 | Down | 7.56E+00 |
|  | Oxidized glutathione | Amino acids | 1.06 | 2.91E-02 | 0.15 | 1.695 | Up | 4.45E+00 |
|  | Saccharopine | Amino acids | 12.18 | 1.87E-07 | -2.00 | 2.346 | Down | 8.29E+00 |
| MMI vs. SMI | Creatine | Amino acids | 0.72 | 4.61E-03 | 0.10 | 7.474 | Up | 2.88E+00 |
|  | gamma-Aminobutyric acid | Amino acids | 0.70 | 2.01E-03 | -0.87 | 1.042 | Down | 4.72E+00 |
|  | L-Arginine | Amino acids | 0.65 | 1.78E-02 | -0.36 | 2.051 | Down | 7.70E+00 |
|  | L-Isoleucine | Amino acids | 1.41 | 1.07E-03 | -0.52 | 6.765 | Down | 2.76E+00 |
|  | L-Pipecolic acid | Amino acids | 0.63 | 1.54E-03 | -0.41 | 2.929 | Down | 3.30E+00 |
|  | L-Proline | Amino acids | 0.75 | 1.81E-03 | -0.42 | 2.805 | Down | 3.09E+00 |
|  | L-Tyrosine | Amino acids | 1.13 | 8.62E-03 | -0.46 | 4.071 | Down | 3.94E+00 |
|  | L-Valine | Amino acids | 0.81 | 9.13E-03 | -0.39 | 2.503 | Down | 4.43E+00 |
|  | Oxidized glutathione | Amino acids | 1.06 | 3.42E-02 | 0.14 | 2.611 | Up | 5.63E+00 |
| STR vs. MMI | L-Arginine | Amino acids | 0.65 | 8.04E-03 | -0.38 | 1.634 | Down | 7.70E+00 |
|  | L-Aspartic Acid | Amino acids | 0.70 | 6.68E-04 | -0.74 | 1.757 | Down | 6.23E+00 |
|  | L-Histidine | Amino acids | 0.63 | 2.25E-02 | -0.40 | 1.361 | Down | 7.60E+00 |
|  | L-Proline | Amino acids | 0.75 | 4.22E-02 | -0.36 | 1.621 | Down | 3.09E+00 |
|  | L-Valine | Amino acids | 0.81 | 4.74E-02 | -0.23 | 1.268 | Down | 4.43E+00 |
|  | N-Acetyl-L-phenylalanine | Amino acids | 4.96 | 1.16E-05 | -3.60 | 1.205 | Down | 7.56E+00 |
|  | Phosphoserine | Amino acids | 0.70 | 2.65E-05 | -1.08 | 1.057 | Down | 8.17E+00 |
|  | Saccharopine | Amino acids | 12.18 | 3.40E-06 | -1.52 | 2.421 | Down | 8.29E+00 |

CON, control; STR, strangulation death; MMI, mild myocardial infarction; SMI, severe myocardial infarction.

**
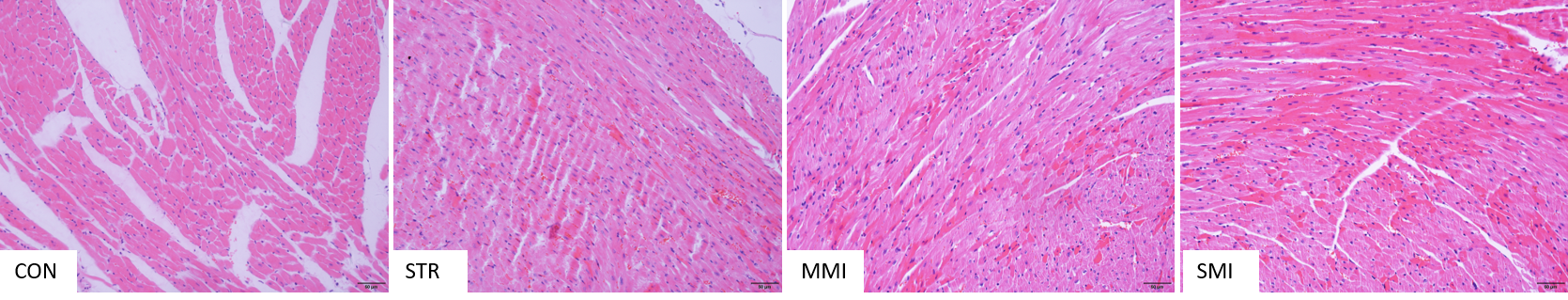
**

**Supplementary Figure S1**

The autopsy findings of mice hearts with HE staining. CON, control; STR, strangulation death; MMI, mild myocardial infarction; SMI, severe myocardial infarction.

**
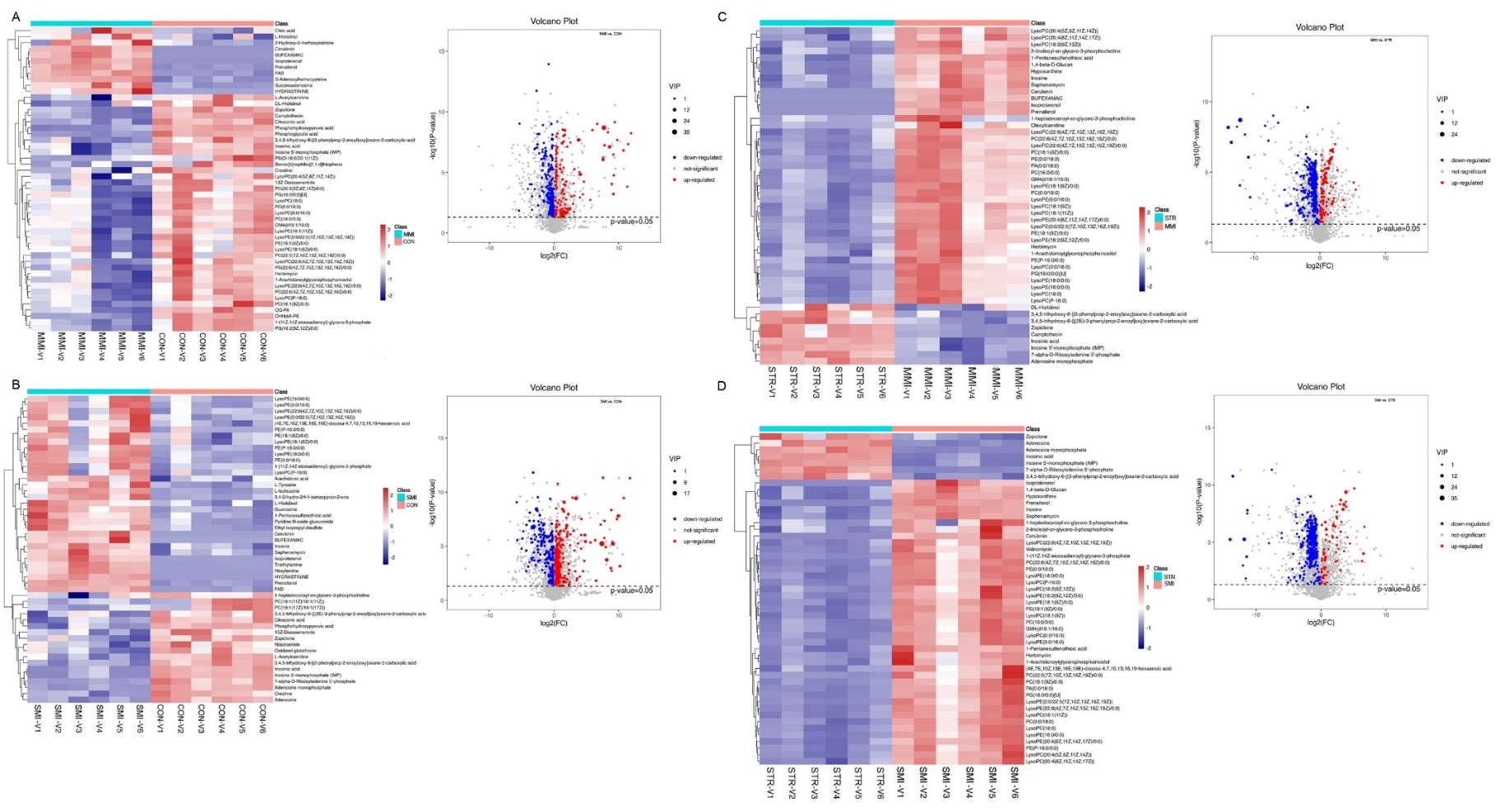
**

**Supplementary Figure S2**

Volcano plots and heatmaps. A: Volcano plot and heatmap in CON vs. MMI group; B: Volcano plot and heatmap in CON vs. SMI; C: Volcano plot and heatmap in STR vs. MMI group; D: Volcano plot and heatmap in STR vs. SMI group. R language “limma” package (http://www.bioconduct or.org/packages/release/bioc/html/limma.html) was applied to identify diferentially expressed genes. CON, control; STR, strangulation death; MMI, mild myocardial infarction; SMI, severe myocardial infarction.

**
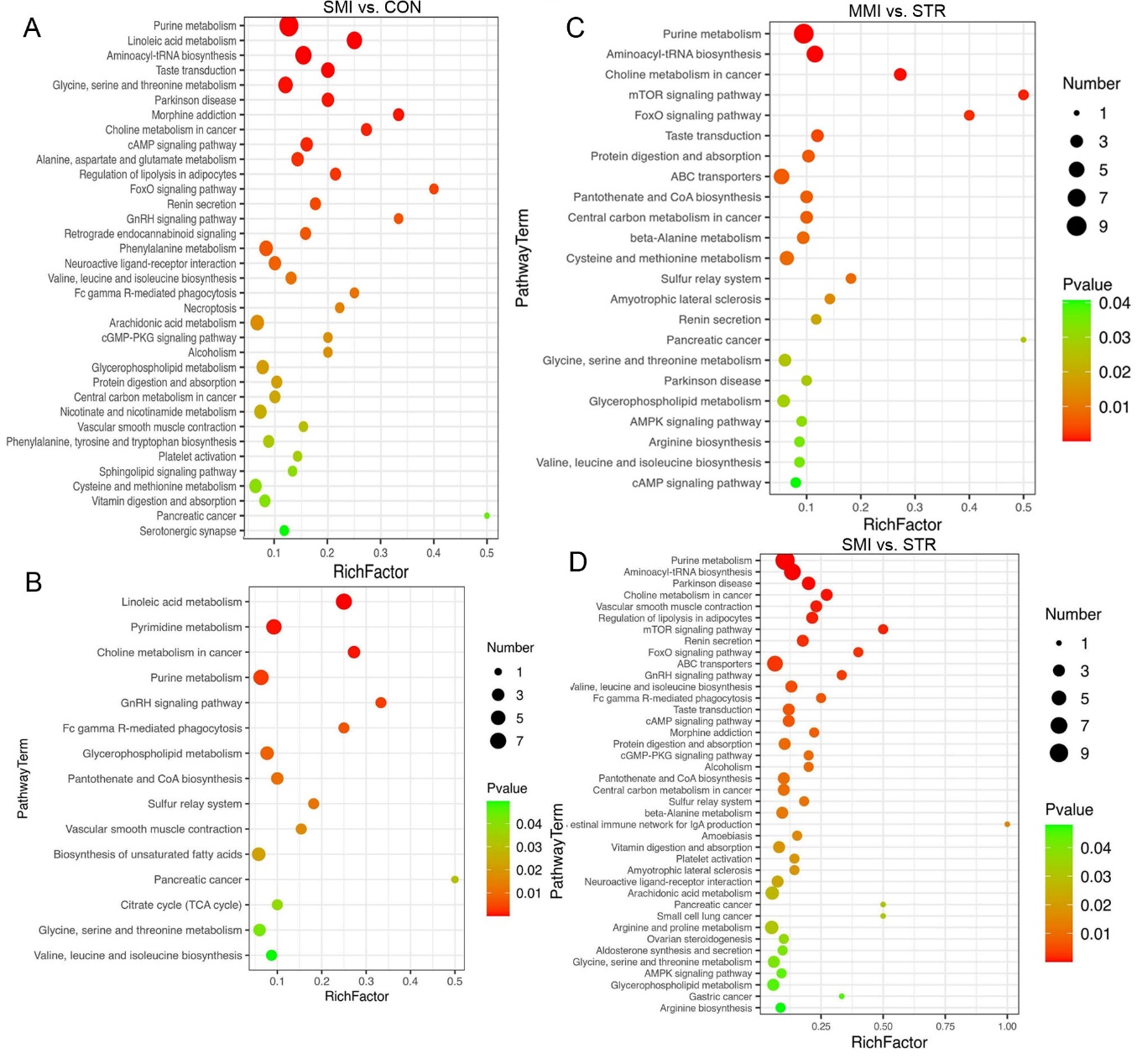
**

**Supplementary Figure S3**

Bubble chart. A: Bubble chart in CON vs. SMI group; B: Bubble chart in CON vs. MMI group; C: Bubble chart in STR vs. MMI group; D: Bubble chart in STR vs. SMI group. CON, control; STR, strangulation death; MMI, mild myocardial infarction; SMI, severe myocardial infarction.


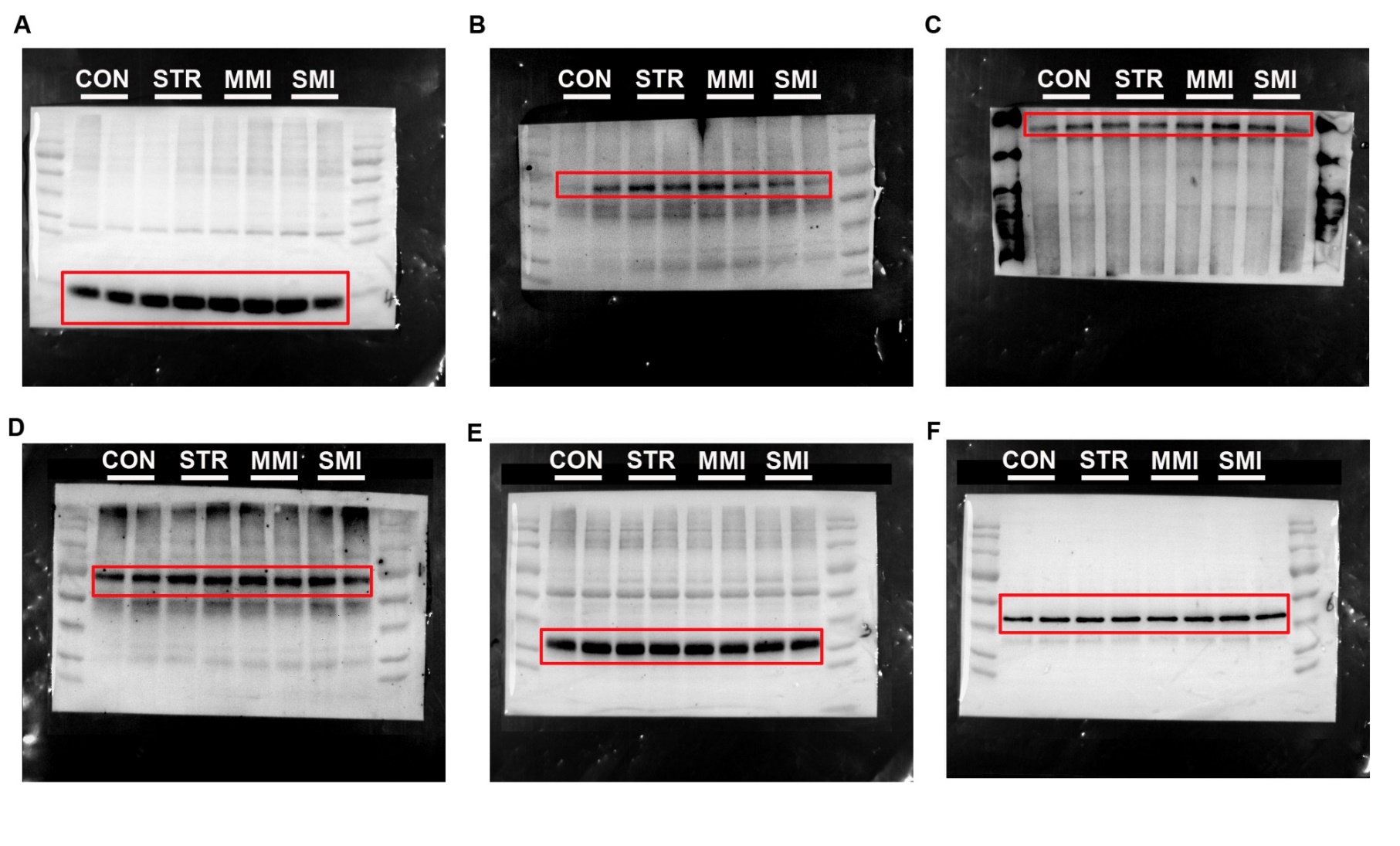


**Supplemental Figure S4**: Full-length blots, used to create figure 6B in the manuscript. A, western blot of Cyt-C; B, western blot of AMPKa1; C, western blot of mTOR; D, western blot of S6K1; E, western blot of PPM1K; F, western blot of β-action. Protein extracts were obtained from the left ventricular wall myocardial with a mammalian protein extraction reagent (Pierce, ThermoScientific, Rockford, IL, USA); The protein extracts of 50 µg were separated by SDS-PAGE under reducing conditions and electrophoretically transferred onto a polyvinylidene fluoride membrane; Proteins of interest were detected with specific antibodies: anti-AMPKα1, anti- Cyt-C, anti-mTOR, anti-PPM1K, and anti-S6K1 at 1:1000 dilution each; Then incubated with a re-probed with horseradish peroxidase-labeled secondary antibody coupled with labeled chemiluminescence or fluorescent molecule, visualized all band intensities; Thereafter, the protein was detected using SuperSignal substrate. Outlined red boxes indicate the cropped regions denoted in figure 6B. CON, control; STR, strangulation death; MMI, mild myocardial infarction; SMI, severe myocardial infarction.
